# Multiple sclerosis: relationship between locus of control and quality of life in persons with low versus high disability

**DOI:** 10.1080/21642850.2022.2050373

**Published:** 2022-03-17

**Authors:** Judith Bijoux Leist, Thomas P. Leist

**Affiliations:** a Department of Counseling Psychology, West Chester University, West Chester, PA, USA; b Comprehensive MS Center, Thomas Jefferson University, Philadelphia, PA, USA

**Keywords:** Multiple sclerosis, disability, locus of control, quality of life, chronic disease

## Abstract

**Background:** Health Locus of Control (HLOC) is the degree to which individuals believe that their health outcomes are controlled by ‘external’ factors – environmental forces, chance, fate, other people, or some higher power – or by ‘internal’ factors – their own behavior or action. Most of the literature on HLOC associates an Internal Health Locus of Control (IHLOC) to pro-health behaviors and better health outcomes. However, a few studies also suggest that in chronic illnesses, an External Health Locus of Control (EHLOC) could be beneficial with respect to pro-health behaviors and perceptions of Quality of Life (QoL), challenging assumptions about what leads to the most effective psychological coping in the face of difficult circumstances. Multiple sclerosis (MS) is a chronic immune condition of the central nervous system and the most frequent cause of non-traumatic disability in young adults, often despite treatment. **Method:** The primary goal of this non-experimental, cross-sectional, quantitative study of 89 individuals with MS was to explore the HLOC of individuals with MS, and to identify whether holding an EHLOC positively impacts the MS patients’ perceived QoL while taking into consideration their level of disability. **Results:** This research found that individuals with higher disability scores tended to hold more EHLOC beliefs, and that there was a significant correlation between QoL and holding EHLOC beliefs. **Conclusion:** This study was able to capture the importance of control beliefs in the QoL of individuals with MS with higher disability. The clinical implications of the findingare explored and areas for further research are suggested.

## Introduction

### Multiple sclerosis

It is becoming more and more evident that biological and psychological factors interact in both directions to contribute to an individual’s overall health. Therefore, mental health providers are often involved in providing help for individuals suffering from chronic physical illnesses, addressing areas of emotional distress often associated with these illnesses and helping patients to increase their ability to embrace medical regimens and to change their lifestyles. Multiple sclerosis (MS) is one such chronic illness. MS is a chronic and progressive disease affecting the central nervous system and affects up to a million individuals in the United States and more than 2.1 million individuals worldwide (National Multiple Sclerosis Society). Individuals are often diagnosed during their most productive years, between 20 and 40 years of age, and it is more prevalent in women (Dobson & Giovannoni, 2018; Ford, 2020). The clinical manifestations of MS are numerous and typically correspond to the area of the central nervous system (CNS) that is affected (Dobson & Giovannoni, 2018). The course of the disease is unpredictable, and many individuals living with MS develop some disability over the course of their illness, including disorganization of motor functions, sensory symptoms, affected vision, and cognitive impairment (Hall, 2010). The complexity of its physical manifestations and the unpredictability of its clinical course are often accompanied by depression (Randolph, Arnett, & Freske, 2004; McNulty, Livneh, & Wilson, 2004; Mohr & Cox, 2001; Turner, Williams, Bowen, Kivlahan, & Haselkorn, 2006), anxiety (Burns, Siddique, Fokuo, & Mohr, 2010; Maurelli et al., 1992; Montel & Bungener, 2007), psycho-social difficulties – such as troubles with self-image (Edmonds, Vivat, Burman, Silber, & Higginson, 2007; Pakenham, 2006), isolation (Arnett & Randolph, 2006), and relationships (Cristall, 1992; Edmonds et al., 2007; Pakenham, 2006) – that can require mental health support.

### Locus of control

Certain inherent characteristics of individuals can either protect them from or make them more vulnerable to severe psychological distress in the face of difficult life circumstances. Among these is the personality trait known as Locus of Control (LOC). An External LOC (ELOC) names the tendency for individuals to attribute the causality of events in their lives to forces outside of themselves, such as luck, fate, or other people; an Internal LOC (ILOC), by contrast, names individuals’ tendencies to attribute causality to their own actions, and, by extension, to believe in their own capacity to control certain outcomes in their lives (Rotter, 1966; Wallston & Wallston, 1978; Wallston et al., 1992). An area in which LOC has been studied extensively in the fostering health-promoting behavior. Scales have been developed to measure the Health Locus of Control (HLOC). Health care providers have tended to view an Internal HLOC (IHLOC) as more favorable as it has been associated with a higher degree of health-promoting behaviors such as exercise, healthy diet, regular checkups, and adherence to medical regimes (DeVito, Bogdanowicz, & Reznikoff, 1982; Graffeo & Silvestri, 2006; Newton & Keenan, 1999; Spector & O'Connell, 1994; Watson, Greer, Pruyn, & Van den Borne, 1990). When faced by chronic or terminal illness, however, things can be more complicated as individuals may no longer have the reinforcement that supports their sense that changes in their behavior will affect their health outcomes. Moreover, research suggests that under such conditions, the LOC can be adaptable, and that an External HLOC (EHLOC) can in fact be beneficial, challenging assumptions as to what leads to the most effective psychological coping when faced with difficult circumstances (Bussing, Ostermann, Neugebauer, & Heusser, 2010; Millet, 2005; Schroder et al., 2007). For example, Wallston (1992) found that individuals with a chronic illness and an IHLOC were at risk of exhibiting and an inflated sense of self-mastery and refuse to seek appropriate help. Individuals with chronic illnesses and an EHLOC, by contrast, were more likely to follow medical staff’s recommendations and – as in a study of cancer patients undergoing chemotherapy – exhibited a greater capacity to relax this aiding biofeedback processes (Burish et al., 1984). Research has also found that religious faith on the part of individuals with an EHLOC correlated with positive physical and mental health outcomes (Fiori, Brown, Cortina, & Antonucci, 2006).

### Health Locus of Control and chronic illness

Researchers have studied the relationship between HLOC and chronic illness in an number of health conditions including diabetes and hypertension (Omeje & Nebo, 2011; Wallston, Wallston, Kaplan, & Maides, 1976), tinnitus (Sirois, Davis, & Morgan, 2006), epilepsy (Asadi-Pooya, Schilling, Glosser, Tracy, & Sperling, 2007), end-stage renal disease (Christensen, Turner, Smith, Holman, & Gregory, 1991), HIV (Burns, Maniss, Young, & Gaubatz, 2005), and cancer (Taylor, Lichtman, & Wood, 1984). While many of these studies have found positive correlations between IHLOC and Quality of Life (QoL), some have also found benefits of an EHLOC and put in question the clear-cut distinction between IHLOC and EHLOC in the context of chronic illness. There is a paucity of research on HLOC and MS. In a study that explored the relationship between LOC, anxiety, and depression found that a shift from an internal to an external orientation tended to occur over the course of the disease, with 88.6% of individuals diagnosed for more than 10 years having come to hold an ELOC (Vuger-Kovacic, Gregurek, Kovačić, Vuger, & Kalenić, 2007).

The present study further explores the relationship between HLOC and QoL in the case of chronic illnesses through a focused study of individuals with MS and different levels of disability. Two research questions were explored: (1) Are individuals with MS and higher levels of disability more likely to hold an EHLOC than those with lower levels of disability? (2) Do individuals with MS who scored high on EHLOC endorse better QoL?

## Methods

### Design

This study employed a non-experimental quantitative design to investigate the relationship between Locus of Control (LOC) and Quality of Life (QoL) in patients with MS as a function of the level of disability and examined the relationship between HLOC beliefs, level of disability, and QoL. Health Locus of Control (HLOC) was measured by the Multidimensional Health Locus of Control Form C (MHLC-C) (Wallston, Stein, & Smith, 1994). The level of disability was assessed by the self-administered phone questionnaire of the Expanding Disability Status Scale (EDSS) – validated self-administered-phone form (Bowen, Gibbons, Gianas, & Kraft, 2001; Kurtzke, 1983). QoL in individuals with MS was measured with the MS Quality of Life Scale (MSQoL-54) (Vickrey, Hays, Harooni, Myers, & Ellison, 1995).

### Participants

A total of 102 individuals who self-identified as having MS consented to participate in the study. Eighty (78.4%) of these were recruited from the Comprehensive Multiple Sclerosis Center at Thomas Jefferson University in Philadelphia, Pennsylvania and were known to meet diagnostic criteria for MS (Thompson et al., 2018). Twenty-six individuals responded to a posting on the website of the National MS Society Website and 22 of these (21.6% of the study population) self-identified as having MS and volunteered to participate in the study. Of the 102 completed questionnaires, 13 that missed three or more questions on any of the measures were excluded from analysis by listwise deletion strategy. The data from 89 individuals (65 women, 24 men) were included in the study. The age of participants ranged from 30 to 65 (mean 45.75 ± 9.13 years). Fifty-nine self-identified as Caucasian (66%), 24 as African American (27%), three as Asian (3.4%), one as Hispanic (1.1%), one as multiracial (1.1%), and one did not disclose an ethnicity (1.1%).

### Measures

#### Expanded Disability Status Score (EDSS)

This rating system was specifically designed to classify the MS patient’s level of disability and was used together with the demographic form at the outset of the study in order to determine whether patients meet inclusion criteria. EDSS is based on the assessment of bodily functions that are controlled by the Central Nervous System, and it is the most widely used assessment tool in MS research (National Multiple Sclerosis Society). For example, question #3 measures mobility: ‘Are you able to walk without help?’ The EDSS yields a score that ranges from 1 to 10; the higher the EDSS score, the more profound the patient’s disability level (Kurtzke, 1983). An instrument has been developed to allow self-administered assessment of the EDSS. The inter-rater reliability between the standard physician administered and the self-administered EDSS is high (Pearson *r* = 0.90) (Bowen et al., 2001). In the current study, this self-administered version of the test was used and to divide study participants into two groups: no or minimal disability (EDSS ≤ 3.5) and moderate to high disability (≥ 4) (European Medicines Agency, Guideline on clinical investigation of medicinal products for the treatment of Multiple Sclerosis, 2015).

#### Multidimensional Health Locus of Control (MHLC)

Inspired by Rotter’s Locus of Control scale, Wallston et al. (1976) developed the Health Locus of Control (HLOC) scale. This scale was later revised and expanded and is known as the Multidimensional Health Locus of Control (MHLC) scale. It assesses specific beliefs about control over health (Wallston, Wallston, & De Vellis, 1978). Currently, there are three forms of the MHLC scales. Form C of the MHLC scale is an 18 items, general purpose, condition-specific locus of control scale adaptable for use in medical or health-related conditions (Wallston et al., 1994). The items on the MHLC-C are divided into internality subscales (6 items), chance externality (6 items), and powerful other externality. Patients are asked to rate on a 6-point Likert scale the degree to which they agree or disagree with each health belief statement. The score for each subscale ranges from 6 to 36 indicating the strength of the belief: internality (Wallston et al., 1994).

#### MS Quality of Life Instrument (MSQoL-54)

According to the World Health Organization (WHO), Quality of Life (QoL) is defined as ‘an individuals’ perceptions of their position in life in the context of the culture and value systems in which they live and in relation to their goals, expectations, standards and concerns’ (World Health Organization, 1997, p. 1). The MS Quality of Life Instrument (MSQoL-54) was used to assess the perceived health-related QoL (Vickrey et al., 1995). The MSQoL-54 covers a broad range of MS-specific domains and assesses the impact of the condition on the individual’s well-being. The MSQoL-54 is a structured self-report questionnaire that contains 54 questions measuring 12 areas that are grouped into 12 subscales: physical function, role limitation (physical), role limitation (emotional), pain, emotional well-being, energy, health perception, social function, cognitive function, health distress, overall quality of life and sexual function. The scores are computed into two summary scores: the mental health composite summary and the physical health composite summary.

The following is sample questions from a few of the scales. The physical functions scale asks, for example:

Does your health limit you in these activities? If so, how much? Climbing several flights of stairs: _Yes, limited a lot; _Yes, limited a little; _No, not limited at all.

The sexual function scale asks, for example:

How much of a problem was each of the following for you during the past 4 weeks?

Male: Difficulty getting or keeping an erection?

Female: Inadequate lubrication?

The emotional well-being scale asks, for example:

How much of the time during the past 4 weeks have you felt so down in the dumps that nothing could cheer you up?

The health distress scale asks, for example:

How much of the time during the past 4 weeks were you discouraged by your health problem. _All of the time; _ Most of the time; _A good bit of the time; _Some of the time; _A little of the time; _ None of the time

The cognitive function scale asks, for example:

Have others, such as family members or friends, noticed that you have trouble with your memory or problems with concentration?

The scores for the subscales range from 0 to 100.

### Data collection

Data collection consisted of the administration of the demographic form and the EDSS-self-administered-phone form followed by the 12 scales of the MSQoL-54 and then MHLC. The MSQoL-54 was administered before the MHLC to control for a potential order effects of reflection on LOC on the MSQoL-54. Data were collected at the Comprehensive MS Center in a private room or at the participants’ own homes. After completion of the questionnaires, participants were encouraged to ask questions or to voice concerns about the process. After completing the questionnaires, a debriefing form was read and given to study participants. Included in this form was the recommendation to contact the social worker at the Comprehensive MS center or to visit their nearest emergency room as needed for emotional disturbances arising during the process of answering the questionnaires.

### Data analysis

Descriptive statistics, including means and standard deviations for continuous variables or frequencies and percentages for categorical variables, were calculated for all study variables. Disability scores (EDSS) were dichotomized as ‘low’ disability (≤ 3.5) or ‘high’ disability (>4) (see Table 1 for descriptive statistics). Descriptive statistics for Health Locus of Control (HLOC) were broken down by specific category (see Table 2). Skew and kurtosis assessed for all continuous variables suggested normal distribution. Histograms and normal quantile-quantile plots were also examined for all continuous variables and were also consistent with normality. Means and standard deviations for all QoL subscales are presented in Table 3 overall and by level of disability.

**Table 1.** Mean disability (EDSS) and disability cohorts.

	Mean	SD	*N* (%)	Skew	Kurtosis
EDSS (Total Cohort)	3.8	2.27	89 (100%)	−0.12	−1.4
EDSS ≤4.0 (Low EDSS Cohort)			49 (55.1%)		
EDSS > 4.0 (High EDSS Cohort)			40 (44.9%)

**Table 2.** Mean health locus of control by category.

	Mean	SD	Skew	Kurtosis
HLOC Internal	18.82	6	−0.06	0.063
HLOC External Chance	16.81	5.8	−0.014	−0.98
HLOC External Doctor	13.26	3.4	−0.65	0.081
HLOC External Other	7.26	2.7	0.051	−0.61

**Table 3.** Quality of life and disability levels.

QoL	Overall *M* (SD)	Low disability *M* (SD)	High disability *M* (SD)	*t*-test
Physical Function	10.18 (5.59)	13.67 (3.99)	5.92 (4.13)	*t* = 8.96, df = 87, *p*-value = <.001
Health Perception	9.62 (3.87)	10.60 (3.65)	8.41 (3.84)	*t* = 2.75, df = 87, *p*-value = 0.007
Energy	5.32 (2.33)	5.82 (2.45)	4.71 (2.05)	*t* = 2.27, df = 87, *p*-value = 0.026
Physical Role Limitations	6.47 (5.19)	9.06 (4.13)	3.30 (4.60)	*t* = 6.22, df = 87, *p*-value = <.001
Pain	7.66 (2.98)	8.85 (2.50)	6.20 (2.88)	*t* = 4.65, df = 87, *p*-value = <.001
Sexual Function	5.32 (2.79)	5.82 (2.60)	4.69 (2.91)	*t* = 1.94, df = 87, *p*-value = 0.056
Social Function	8.36 (3.07)	9.38 (2.45)	7.11 (3.31)	*t* = 3.71, df = 87, *p*-value = <.001
Physical Health Distress	6.97 (3.14)	7.51 (2.90)	6.30 (3.31)	*t* = 1.84, df = 87, *p*-value = 0.069
**Physical Health Composite**	60.42 (22.27)	70.75 (18.23)	47.77 (20.30)	*t* = 5.62, df = 87, *p*-value = <.001
Mental Health Distress	8.86 (3.99)	9.56 (3.69)	8.02 (4.21)	*t* = 1.84, df = 87, *p*-value = 0.069
Overall Quality of Life	12.36 (3.91)	13.23 (3.53)	11.29 (4.12)	*t* = 2.39, df = 87, *p*-value = 0.019
Emotional Well-Being	19.91 (5.81)	20.25 (5.95)	19.50 (5.68)	*t* = 0.61, df = 87, *p*-value = 0.545
Emotional Role Limitations	16.49 (9.45)	18.53 (8.17)	13.99 (10.37)	*t* = 2.31, df = 87, *p*-value = 0.023
Cognitive Function	9.86 (4.40)	10.42 (4.45)	9.17 (4.29)	*t* = 1.34, df = 87, *p*-value = 0.185
**Mental Health Composite**	67.48 (22.49)	71.96 (21.22)	61.99 (23.05)	*t* = 2.12, df = 87, *p*-value = 0.037

In addition, the means, and standard deviations for all QoL subscales and composites separated by level of disability were compared. QoL was lower in every domain for participants in the high disability group compared to the low disability group. These groups were compared using independent sample *t*-tests that showed that these differences were in most cases statistically significant. Because of the exploratory nature of this study, sample size, and nature of the underlying assessments results were not adjusted for multiple comparisons.

## Results

### Relationship among variables

Pearson product moment correlations for all continuous study variables were conducted. The correlations between the QoL mental and physical composites with locus of control and disability are shown in Table A1 (in Appendix). Older age, which would be expected to generally correlate with longer disease duration, was associated with moderately higher disability scores (*r* = .35) and with lower physical and sexual function (*r* = –.34 for both). As expected, higher disability scores were correlated with lower QoL. Examining the LOC measures**,** only the EHLOC-Chance showed any correlation with the MSQoL-54 sub scales and composites. EHLOC-Chance correlated with physical function, physical role limitations, sexual function, and social function MSQol, all in the direction of higher HLOC-Chance being associated with better QoL Other bivariate relations between LoC and QoL were not statistically significant. Table 3 shows the means and the standard deviations for all the Quality of Life (QoL) subscales and composites. While not statistically significant, it is important to note that, as a whole, the mental health QoL was higher than the physical health QoL (*M* = 67.48 versus *M* = 60.42).

The first question of the study was whether individuals with higher MS-related disability score higher on External Health Locus of Control (EHLOC) measures. Independent samples *t*-tests were used to compare the means on Internal Health Locus of Control (IHLOC) and EHLOC-Chance subscales in individuals with low versus high disability (Table 4). There was a significant difference between low and high disability groups on EHLOC-Chance (*t* (87) = 2.21, *p *< .030) but not on the IHLOC subscale (*t* (87) = 1.57, *n = *.120). Higher EDSS scores did correlate with higher EHLOC-Chance.

**Table 4.** Means and *T*-tests comparing locus of control by low and high disability.

	Low Disability *M* (SD)	High Disability *M* (SD)	*t*-test
IHLOC	19.71 (6.04)	17.73 (5.82)	*t* (87) = 1.57, *p* = 0.120
EHLOC-Chance	18.00 (6.00)	15.35 (5.14)	*t* (87) = 2.21, *p* = 0.030*

*p = 0.01

The second question of the study was whether higher EHLOC beliefs correlate with better QoL, as measured by the MSQoL-54. The hypothesis two was tested by using Pearson correlation analysis between EHLOC-Chance and the QoL Composites and subscales. The results show that EHLOC-Chance correlated positively with higher values on the physical function QoL (*r* (87) = .22, *p* < .001), physical role limitation Qol (*r* (87) = .23, *p* < .001), sexual function QoL (*r* (87) = .23, *p* < .056), and social function QoL (*r* (87) = .21, *p* < .001). Higher EHLOC beliefs correlated with better QoL (Table A1). This was further supported with a moderation analysis between locus of control, disability and physical quality of life (Table 5).

**Table 5.** Moderation analysis for LoC external other × disability on physical health QoL.

	PhysFunc	HPerc	Energy	RoleLimitPhys	Pain	SexFunc	SocFunc	HDistressPhys	Comp
(Intercept)	19.20***	7.23**	3.24	8.09*	8.77***	10.65***	9.17***	5.99**	76.30***
	(2.93)	(2.67)	(1.65)	(3.19)	(1.82)	(1.95)	(2.07)	(2.19)	(13.99)
Age	−0.11*	0.09*	0.04	−0.01	0.01	−0.10**	−0.02	0.02	−0.13
	(0.05)	(0.05)	(0.03)	(0.05)	(0.03)	(0.03)	(0.04)	(0.04)	(0.24)
Caucasian	0.63	0.13	0.12	1.54	1.35*	0.14	1.07	0.21	4.12
	(0.91)	(0.83)	(0.51)	(1.00)	(0.57)	(0.61)	(0.65)	(0.68)	(4.36)
LoC External Other	−0.18	−0.11	0.07	0.06	−0.16	−0.09	0.03	0.04	−0.37
	(0.20)	(0.18)	(0.11)	(0.22)	(0.13)	(0.13)	(0.14)	(0.15)	(0.97)
High Disability	−7.00**	−1.32	0.81	−5.98*	−0.82	−1.14	−0.03	2.77	−11.21
	(2.52)	(2.30)	(1.41)	(2.75)	(1.57)	(1.67)	(1.78)	(1.89)	(12.02)
LoC External Other x High Disability	−0.05	−0.22	−0.31	0.06	−0.30	0.06	−0.31	−0.61*	−1.70
	(0.33)	(0.30)	(0.19)	(0.36)	(0.21)	(0.22)	(0.24)	(0.25)	(1.60)
*R*^2^	0.51	0.16	0.12	0.33	0.33	0.13	0.19	0.13	0.30
Adj. *R*^2^	0.48	0.10	0.06	0.29	0.29	0.08	0.14	0.08	0.26
Num. obs.	89	89	89	89	89	89	89	89	89

Note: Regression coefficients are shown with standard errors in parentheses.

***p < 0.001.

**p < 0.01.

*p < 0.05.

## Discussion

Health Locus of Control (HLOC) refers to the beliefs that guide individuals’ tendency to attribute their health to their own actions or to external agents. As with the generic Locus of Control (LOC) construct, individuals who believe that by their own actions they have control over their health are considered as having an Internal HLOC (IHLOC), while individuals who believe that external factors, such as medical professionals, luck, or fate, significantly contribute to their health, are considered as having an External HLOC (EHLOC). The literature generally supports the view that an IHLOC correlates with better health outcomes, e.g. in the case of hypertension (Omeje & Nebo, 2011), early diabetes (Wallston et al., 1976), and tinnitus (Sirois et al., 2006). While these findings ‘may be characteristic of physically healthy or acutely ill individuals, there is a body of research suggesting that this is not the case for chronically ill individuals’ (Burish et al., 1984, p. 326), particularly when faced with progressive loss of function and increasing disability. For instance, in epilepsy (Asadi-Pooya et al., 2007), end-stage renal disease following a failed transplant (Christensen et al., 1991), and in HIV/AIDS (Burns et al., 2005), the EHLOC was found to be more beneficial with respect to coping. Studies have also shown the co-existence of IHLOC and EHLOC in chronic illnesses such cancer (Taylor et al., 1984). In MS a shift of the HLOC from IHLOC in the earlier in the disease to EHLOC later in the disease (Vuger-Kovacic et al., 2007). Guided by this latter observation, the current study aimed to better understand whether the HLOC – whether internal or external – correlates with the perception of QoL of individuals with varying degrees of MS-related disability.

### Correlation between higher levels of disability and EHLOC

Individuals with higher disability as measured by the (EDSS) were likely to hold a belief that chance, EHLOC-Chance, played a strong role in their health. The results of this study contribute to the discussion that LOC is not a fixed personality trait but adapts with changing life circumstances and challenges. Vuger-Kovacic et al. (2007) observed in their longitudinal study that individuals who were initially categorized as having an internal LOC endorsed more external beliefs as their condition progressed. DuCette (1974), cited in Wallston et al. (1978), reported that recently diagnosed diabetics who held an IHLOC behaved in a more health-conscious manner than those holding an EHLOC but this difference in behavior was not observed in individuals with longer standing diabetes. Diabetics with longer standing disease and an IHLOC missed more doctors’ appointments and were less likely to adhere to a recommended diet. DuCette (1974) suggested that the unpredictable and uncontrollable aspect of the condition may bring on a change in the attitude concerning control of the health condition. When face with a chronic illness such as MS, patients may deem the disease impact as beyond their control.

In addition, the finding that those with advanced levels of disability are more likely to hold an EHLOC-Chance converges with the findings of a number of studies on the relationship between HLOC and chronic illness noted previously. Studies in cancer (Burish et al., 1984), tinnitus (Sirois et al., 2006), epilepsy (Asadi-Pooya et al., 2007) and failed renal transplants (Christensen et al., 1991) found that individuals were more likely to hold an EHLOC. In their longitudinal study of MS patients, Vuger-Kovacic et al. (2007) found that with the progression of the disease, individuals were likely to shift from an IHLOC to an EHLOC. The results of the current study also support the presence of an EHLOC-Chance with more advanced disease.

### Correlation between EHLOC and QoL

The results of the current study suggest that contrary to much of the literature on LOC holding an EHLOC when living with greater MS-related disability correlated with increased QoL as assessed with the MSQoL-54, which assesses both the physical and the mental health impact of MS. The results suggest a relationship between level of disability, a belief in the power of Chance and a higher QoL. A belief in EHLOC-Chance over the outcomes of one’s health positively correlated with most of the physical QoL measures, namely physical function, physical role limitation, and sexual and social function. This suggests that when individuals believe that factors outside of their own control are responsible for health outcomes, they may be able to better adjust to the disease, perhaps due to the fact that they do not blame themselves for their condition or believe that something they could have done would have changed the course of the illness.

EHLOC-Chance beliefs significantly correlated with physical subscales of the MSQoL-54 but not mental health subscales. This suggests that participants in the study did not believe that Chance was a factor in mental health. For instance, on the Emotional Well-being subscale, two of the questions are: ‘ … have you felt downhearted and blue?’ and ‘Have you felt so down in the dumps that nothing could cheer you up?’ It is hard to imagine that while answering these questions individuals, knowing that their condition may directly or indirectly impact their mood, will believe that forces outside of themselves such as luck or fate are responsible for their mood. Another example of the Cognitive Function scale: ‘Did you have trouble keeping your attention on an activity for long?’ Again, the individual’s perception of his or her cognitive ability would not logically be influenced by a belief such as chance, luck, or fate. Patients are more likely to see these difficulties as an intrinsic part of their condition. But, it is also possible that individuals may hold the belief to be able to influence these particular ailments.

In contrast to the Mental Health QoL scales, an EHLOC-Chance belief positively correlated with many of the Physical Health QoL subscales, including Physical function subscale which includes questions like: ‘Does your health limit you in these activities, and if so, how much?’ ‘ … Moderate activities such as moving a table, pushing a vacuum cleaner, bowling … ’ It appears that presence of limitations that are noticeable by others, the individual with MS is more likely to believe that it was chance, luck or fate that contributed to the reduction in function. The fact that the clinical manifestations of MS are highly variable between individuals may enforce the belief in luck. This may also serve to explain the correlation between EHLOC-Chance and the Physical Role Limitation subscale, which asks questions like: ‘As a result of your physical health did you … cut down on the amount of time you could spend on work or other activities?’ and the correlation between EHLOC-Chance and the Social Function subscale. The latter asks questions like: ‘To what extent have problems with your bowel or bladder function interfered with your normal social activities with family, friends, neighbors, or groups?’ Along the same line, the correlation between EHLOC-Chance and the Sexual Function subscale, which asks questions like: ‘during the past 4 weeks … have you experienced lack of sexual interest … difficulty having orgasm … ability to satisfy sexual partner’ may indicate that patients may interpret disease impact in this area as disease related and subject to the variability of its associated presentation. Based on the results it can be hypothesized that participants appeared to significantly rely on the power of luck and fate as a mechanism to protect themselves against self-blame and to maintain QoL.

While EHLOC-Chance positively correlated with the Physical Health QoL, there were individual subscales of Physical Health QoL where this was not observed. How can one make sense of these discrepancies within the same construct? There are a few plausible explanations worth considering. A closer look at some of these Physical Health subscales shows that they are subjective and rely on an emotional reaction. Two of the questions on the Health Perception subscale ‘I am as healthy as anybody I know’ and ‘I expect my health to get worse.’ Serve to exemplify this. Similarly, on the Health Distress subscale, two of the questions read: ‘Were you frustrated about your health?’ and ‘Did you feel weighed down by your health problem?’ It could therefore be hypothesized that it is not likely that an individual would endorse Chance as a determinant factor in these areas. Another way of explaining the discrepancy, particularly for Pain and Energy/Fatigue (two important areas of MS patients’ experiences), is that these constructs are highly subjective and are also expected by the individual to be an intrinsic part of their condition. There is also the possibility that the characteristics of the study sample itself may contribute to the findings. In this current study, younger and older MS participants were not separated. Research has shown that older individuals with MS are different in their assessment of pain and energy (Molten et al., 2008). Older patients with a longer duration of disease develop higher thresholds for pain and lower their expectation for energy. The mean age of the cohort studied was 45.8 and having a high number of older participants, with longer disease duration can affect the assessed outcome.

Changes in perceived control over one’s health in the face of growing illness or disability in an external direction may lead to a better adjustment to one’s condition or better QoL. Notably, individuals in the study at advanced stages of MS are more likely to hold an EHLOC-Chance and are more likely to report better QoL when they put their belief in the role of Chance in affecting their lives. On this point, the current study converges with three earlier investigations. Burish et al. (1984) found that women who had an EHLOC prior to undergoing chemotherapy were more likely to have positive outcomes to biofeedback treatment prior to chemotherapy. This correlation was maintained with the subsequent course of chemotherapy. In contrast, women with an IHLOC had initial success with biofeedback, but did not retain this response with subsequent treatments. This group also reported more anxiety. The findings of Burish et al.’s (1984) study could be interpreted as indicating that individuals with EHLOC were more likely to give themselves over to the treatment, and to maintain the relaxation required for the success of the biofeedback treatment. Zampieri and Souza (2011) studied the relationship between LOC, depression, and QoL in Parkinson’s disease and found that individuals who scored high on the EHLOC had higher QoL. Burns et al. (2005) reported on the usefulness of EHLOC in predicting mental health QoL in individuals living with HIV/AIDS. These authors found no correlation between IHLOC and mental health QoL.

The results of this study were discordant with findings in the majority of studies on the topic. While other studies support the idea that increasing disability is associated with generally having an EHLOC, holding an ELOC is generally not viewed as fostering positive outcomes. Most studies on LOC in general, and on HLOC in particular, emphasize the positive aspects of holding an IHLOC rather than an EHLOC and emphasize the correlation between an IHLOC and positive health behaviors including adherence to treatment, exercise, and healthy lifestyle choices. Mackey (2002) and Omeje and Nebo (2011) found that an IHLOC correlated with higher levels of smoking cessation, requests for health-related information, and keeping of medical appointments. Asadi-Pooya et al. (2007) found that an IHLOC correlated with greater seizure control among individuals with epilepsy. Sirois et al. (2006) reported that those holding an IHLOC endorsed greater psychological wellbeing. To address the difference in findings between the cited studies and this investigation, it is important to note that these studies assessed the effect of LOC on a health-related behaviors that are objectively measurable, while this research focused on an individual’s self-assessment of QoL, which is not an objectively measurable behavior.

The majority of studies on HLOC that demonstrated that IHLOC correlates with better health behaviors also reported that EHLOC was associated with higher levels of anxiety and depression when assessed with tools including the Hospital and Depression scale (HAD) or Beck Depression Inventory (BDI). These studies, in contrast to this investigation, tend not to focus on individuals’ self-report of QoL. For instance, Asadi-Pooya et al. (2007) reported on the positive relationship between an IHLOC and seizure control used the same MHLOC-C measure as this study, but the assessed correlation between an IHLOC and seizure control, depression, and anxiety with the Hospital and Depression scale (HAD), which is not comparable to the MSQoL-54 used here. While the HAD assesses objective signs of depression and anxiety, the MSQoL-54 evaluates self-reports of mental well-being. For example, an individual who reported low levels of physical QoL also endorsed a high level of mental well-being. The MSQoL-54 used in the present study has the benefit of allowing participants to offer an assessment of their personal perception of their own well-being. The divergence between patients’ assessment of their own mental states and the objective manifestations of their physical QoL warrants further investigation.

The divergent findings can be attributed to the fact that the present study measured different constructs than many of the previous studies on the relationship between QoL and HLOC in individuals with chronic illnesses. Specifically, the findings of this study emphasize individuals’ self-perception of their own mental well-being, which was found to correlate positively with a belief, among individuals with high EDSS, in the powerful role of Chance/Fate in their lives.

### Clinical implications

This study underscored the importance of paying attention to mental health in the overall care of individuals with MS, specifically as it relates to their perceived sense of control. This study was able to capture the importance of control beliefs in the QoL of individuals with MS with higher disability. It suggests that mental health professionals and other members of the disease-specific care team, including physicians, mid-level providers, nurses, and social workers should encourage individuals with MS under their care to express what would reasonably contribute to their quality of life and then assist them in negotiating realistic expectations for care. It is likely that psychologists and other mental health professionals in general practice may be directly involved in providing psychological care to an individual with MS. In such a situation, it would be useful to assess the individual’s perceived LOC and more importantly the individual’s allocation of their QoL, and to identify the factors that may decrease the individual ability to adjust to the challenges of their chronic condition. Because of the meaning, individuals attribute to both their sense of control and QoL, the clinician should create an environment that allows clients to express their needs and find the support to explore their choice of appropriate coping strategies that help them feel more competent in their daily lives.

### Limitations

Several factors limit the interpretation of the results of this study. The analyses were based on self-reported data: while, as stated, there are significant benefits to studying individuals’ self-reports concerning their QoL, self-reports can also be intentionally or unintentionally biased. In addition, some informative questions that might affect the interpretation of results were not included in the questionnaire. For example, the duration of the disease was not assessed as part of the questionnaire; however, the point of time at which beliefs about control are assessed may be significant to the understanding of adaptation to a progressively disabling condition. In addition, the questionnaire did not record the type of current or prior MS modifying therapy or concurrent medications, which could be a moderating factor in our analysis. Further, depression, fatigue, and cognition were not separately assessed.

The specificities of the places of recruitment also pose limits to the results of the study. Recruited predominantly from the Comprehensive Multiple Sclerosis Center at Thomas Jefferson University located in Center City Philadelphia, the sample was not comparative to the national population of MS subjects. For instance, African Americans comprised 27% of the study participants but represent only 8% of the MS population nationally (http://www.nationalmssociety.org/index.aspx). Philadelphia has a significant African American population, explaining the higher percentage of this group in the study. Therefore, the results of this study may not adequately generalize to other settings or to different areas of the country.

While this discrepancy may limit the generalization of the study, it can also be viewed as strength as it gave a stronger voice to a minority group who is often underrepresented in studies and may provide valuable information pertaining to African Americans. Furthermore, the method of recruitment may have inadvertently biased the sample. Many of the participants from the MS Center were enrolled when they came for their infusion of disease modifying therapy, suggesting that they were probably having more advanced disability. Being on infusion with a disease modifying therapy is usually a sign of a more aggressive form of MS, and it is reasonable to assume that individuals who volunteered from this group were more impacted by the condition.

The HLOC questionnaire has some limitations, for instance, no significant correlations were discovered between overall LOC and QoL. This fact suggests that taken alone, LOC does not predict the individual’s assessment of their QoL. And the construct may not capture whether individuals living with worsening MS grow to understand the disease process as an entity by itself that is not subject to outside factors of luck, fate, or intervention by others.

As a correlational study, causation cannot be inferred from the present data alone. Furthermore, qualitative studies with this population are needed to explain the relationship between LOC and QoL and for a better understanding of people’s experience with MS. Also important are longitudinal studies that follow individuals with MS from diagnosis, with different questionnaires at different stages of the disease, in order to assess how LOC might change over the course of the disease. Additionally, the statistical analysis was not correct for multiple comparisons.

### Directions for further research

The present study indicates that researchers should not rely on single measures of mental health when assessing individuals’ well-being and QoL, as different measures may measure different aspects of individuals’ reality. It seems important to include individuals’ self-assessments of their own mental health, such as the MSQoL-54, alongside other measures, such as a Beck Depression Inventory or Beck Anxiety Inventory that are viewed as less subjective.

Due to the stated limitations, a mixed method study, which included a phenomenological or other qualitative approach, would be highly beneficial to better understand contextual factors such as individuals’ experience of and adjustment to their disease, and the role that LOC plays in these areas. There are surely multiple factors, such as psychological makeup and social as well as environmental factors, family support, education, and socioeconomic status to mention just a few, that contribute to the overall perception of QoL of an individual facing MS. It is important to understand these variables in terms of the individual’s own perception of what impacts their lives. Many of the study’s participants were very eager to discuss their experience in their own words. This was very much evident both in conversations shared between the researcher and some of the participants while obtaining consent, and in the special notes that many of the participants wrote next to some of the questions in the MSQoL-54. Many came back with add-on narratives in addition to the choice of degree made available on the questionnaire. No one questionnaire could really capture the participants’ experiences and perceptions, hence, qualitative components of studies in future research are needed to capture the participants’ perceptions and to help understand how MS and LOC have influenced QoL.

Finally, while not in the scope of this present study, certain observations made during the data collection process strongly suggest that gender may affect the HLOC beliefs. Men and women differ in their self-assessment of their quality of life (Pudrovska, 2015) therefore further research into gender differences in adjustment to MS is suggested. In addition, the statistics used in this study may have not been able to capture the impact of EHLOC-Other on QoL and further analyses are suggested. Furthermore, it may also be of value to research the impact of ethnicity on perceived control and QoL. It may be of value to study the relationship between locus of control and resilience (Coutu, 2002; Dunn, Uswatte, & Elliott, 2009; Hariharan, Karimi, & Kishore, 2014; Schure, Odden, & Groins, 2013) in individuals living with MS and with high disability, particularly to explore if the direction of a person’s beliefs about control has an impact on their capacity to be resilient in the face of a disabling condition.

## Appendix

**Table A1.** Pearson correlation.

	1.	2.	3.	4.	5.	6.	7.	8.	9.	10.	11.	12.	13.	14.	15.	16.	17.	18.	19.	20.	21.
1. Age	–	0.35***	−0.10	−0.17	−0.09	−0.20	−0.34**	0.14	0.10	−0.17	−0.05	−0.34**	−0.12	0.03	−0.17	0.03	−0.17	0.04	−0.03	0.06	−0.02
2. EDSS		–	−0.17	−0.14	−0.14	−0.15	−0.76***	−0.32**	−0.36***	−0.61***	−0.51***	−0.29**	−0.47***	−0.30**	−0.61***	−0.30**	−0.34**	−0.16	−0.34**	−0.23*	−0.34**
3. LoC Internal			–	0.24*	0.25*	0.28**	0.07	0.11	−0.03	0.04	0.02	0.01	0.01	0.04	0.04	0.04	0.04	−0.02	0.00	0.03	0.02
4. LoC External Chance				–	0.10	0.33**	0.22*	0.10	0.17	0.23*	0.12	0.23*	0.21*	−0.01	0.20	−0.01	0.07	0.01	0.15	0.16	0.11
5. LoC External Doctor					–	0.16	0.10	−0.14	0.11	0.14	−0.03	−0.14	0.08	0.02	0.06	0.02	0.07	−0.06	0.11	−0.02	0.04
6. LoC External Other						–	0.06	−0.11	−0.03	0.13	−0.17	0.02	−0.01	−0.12	−0.02	−0.12	−0.03	−0.10	0.00	−0.08	−0.07
7. Physical Function							–	0.45***	0.47***	0.76***	0.62***	0.42***	0.61***	0.42***	0.81***	0.42***	0.48***	0.29**	0.46***	0.28**	0.48***
8. Health Perception								–	0.52***	0.44***	0.56***	0.27*	0.58***	0.65***	0.72***	0.65***	0.53***	0.43***	0.47***	0.40***	0.59***
9. Energy									–	0.49***	0.57***	0.23*	0.63***	0.61***	0.72***	0.61***	0.57***	0.61***	0.54***	0.41***	0.67***
10. Physical Role Limitations										–	0.65***	0.34***	0.69***	0.46***	0.81***	0.46***	0.40***	0.26*	0.50***	0.29**	0.49***
11. Pain											–	0.38***	0.71***	0.57***	0.80***	0.57***	0.48***	0.44***	0.43***	0.46***	0.57***
12. Sexual Function												–	0.40***	0.26*	0.49***	0.26*	0.40***	0.40***	0.37***	0.36***	0.45***
13. Social Function													–	0.67***	0.87***	0.67***	0.56***	0.51***	0.63***	0.51***	0.71***
14. Phys. Health Distress														–	0.74***	1.00***	0.65***	0.61***	0.60***	0.46***	0.79***
15. Physical Composite															–	0.74***	0.66***	0.49***	0.65***	0.47***	0.74***
16. Mental Health Distress																–	0.65***	0.61***	0.60***	0.46***	0.79***
17. Overall Quality																	–	0.72***	0.63***	0.40***	0.81***
18. Emotional Well-Being																		–	0.58***	0.53***	0.84***
19. Emotional Role Limitations																			–	0.49***	0.88***
20. Cognitive Functions																				–	0.69***
21. Mental Health Composite																					–

**p* < .05, ***p* < .01, ****p* < .001.
